# Separation, Identification, and Antioxidant Activity of Polyphenols from Lotus Seed Epicarp

**DOI:** 10.3390/molecules24214007

**Published:** 2019-11-05

**Authors:** Zhili Ma, Yi Huang, Wen Huang, Xi Feng, Fang Yang, Deyuan Li

**Affiliations:** 1 School of Laboratory Medicine, Hubei University of Chinese Medicine, Wuhan, Hubei 430065, China; mazhilitoo@hotmail.com; 2 College of Food Science and Technology, Huazhong Agricultural University, Wuhan, Hubei 430070, China; mariahuang@webmail.hzau.edu.cn (Y.H.); huangwen@mail.hzau.edu.cn (W.H.); 3 Department of Nutrition, Food Science and Packaging, California State University, San Jose, CA 95192, USA; xi.feng@sjsu.edu

**Keywords:** lotus seed epicarp, polyphenols, separation, identification, antioxidant activity

## Abstract

Lotus seed epicarp, the main by-product of lotus seed processing, is abundant in polyphenols. In this study, polyphenols in lotus seed epicarp were separated by Sephadex LH-20 gel filtration chromatography to yield Fraction-I (F-I), Fraction-II (F-II), and Fraction-III (F-III). The polyphenol compounds in the three fractions were identified by UPLC-MI-TOF-MS. Six kinds of polyphenol compounds including cyanidin-3-*O*-glucoside, procyanidin trimer, and phlorizin were identified in F-I, and prodelphinidin dimer B, procyanidin dimer, and quercetin hexoside isomer were found in F-II. However, there was only procyanidin dimer identified in F-III. The in vitro antioxidant activities of the three fractions were also investigated. We found F-I, F-II, and F-III had strong potential antioxidant activities in the order of F-III > F-II > F-I. Our results suggested that polyphenols from lotus seed epicarp might be suitable for use as a potential food additive.

## 1. Introduction

Lotus (*Nelumbo nucifera* Gaertn.) is an important aquatic economic crop in China, and it has been widely cultivated in Asia [1,2]. Almost every part of lotus (roots, leaves, flowers, and seeds) not only can be used as a food-stuff, but also have multiple medicinal functions [3]. In China, lotus leaves and seeds are publicly identified as both food and medicine resources. There is much research concerned with lotus leaves and seeds [4,5,6,7]. It has been reported that the extract from lotus leaves and seeds have multiple bioactivities, such as being anti-oxidant [8,9], anti-inflammatory [10], immuno-modulatory [11], and anti-obesity [12] etc. The bioactivities of lotus are due to the functional components in lotus including polysaccharides, polyphenols, flavonoids etc.

From the view of converting food waste into by-products, several kinds of bioactive flavonoid, proanthocyanidin, and phenolic compounds have been extracted and identified from the receptaculum nelumbinis [13,14,15]. As an inedible part of lotus, lotus seed epicarp also has attracted additional attention. Lotus seed epicarp, the main by-product of lotus seed processing, is also abundant in polyphenols and worthy to be used as a functional food. Lotus seed epicarp extract has been used as a potential antioxidant and anti-obesity additive in Chinese Cantonese Sausage, which was the first use of lotus seed epicarp extract in meat product [16]. Liu, et al. [17] reported that polyphenols from lotus seed epicarp at three different ripening stages have good anti-radical abilities, and they identified some phenolic compounds from lotus seed epicarp. Recently, Liu, et al. [18] found that the anti-radical activity of polyphenols from lotus seed epicarp increases by probiotic bacteria bioconversion. Yan, et al. [19] reported that polyphenols from lotus seed epicarp have antiproliferation ability on human hepatoma G2 (HepG2) cells. However, little is known about the purification and identification of the key bioactive polyphenols from lotus seed epicarp.

The aims of this study were to separate polyphenols from lotus seed epicarp into different fractions, characterize the phenolic profiles of polyphenol fractions by UPLC-MI-TOF-MS. The antioxidant activities of polyphenol fractions were also determined.

## 2. Results and Discussion

### 2.1. Purification and Identification of Lotus Seed Epicarp Polyphenols

**Figure 1:**
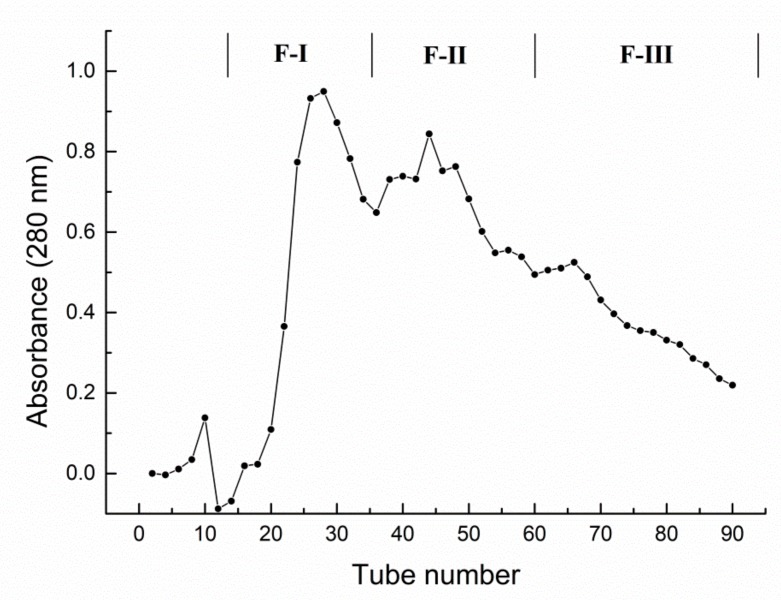
Separation of polyphenols from lotus seed epicarp by Sephadex LH-20 gel column into Fraction-I (F-I), Fraction-II (F-II), and Fraction-III (F-III).

As shown in Figure 1, the lotus seed epicarp polyphenols obtained after AB-8 macroporous resin were then separated with a Sephadex LH-20 gel filtration chromatography and three fractions (Fraction-I (F-I), Fraction-II (F-II), and Fraction-III (F-III)) were separately collected. In order to figure out the structure of the main polyphenols in the three fractions, UPLC-MS/MS was employed to identify the polyphenol compounds.

A total of six phenolic compounds were identified in the three selected fractions. However, Liu, Ma, Ibrahim, Li, Yang and Huang [17] identified four kinds of polyphenols from lotus seed epicarp, and Yan, Luo, Cong, Zhang, Ma and Duan [19] found eight kinds of polyphenols from fresh lotus seed epicarp. These differences may be caused by varied purification and identification methods. Three kinds of polyphenol compounds were identified from the sample of F-I, which were cyanidin-3-*O*-glucoside, procyanidin trimer and phlorizin. As shown in Table 1, compound **1** (RT = 4.68 min) showed a molecular ion ([M−H]^−^) at mass/charge ratio (m/z) of 449.1087. The main fragment at m/z of 287.0558 was identified as cyanidin by the neutral loss of a dehydrated glucose from compound **1** [20]. The result was consistent with the report of cyanidin-3-O-glucoside by Tian, et al. [21]. Compound **2** showed the molecular ion ([M−H]^−^) at m/z of 865.1975 and two fragments at m/z of 449.1088, 287.0557, which was the result of its direct cleavage of the inter flavanic bond, and was identified as the procyanidin trimer [22]. Compound **3** (RT = 7.75 min) showed a molecular ion ([M−H]^−^) at m/z of 435.1293, and the main fragment at m/z of 273.0761 was phloretin by the loss of glucose from compound **3**. Therefore, compound **3** was identified as phlorizin [23].

Three major polyphenol compounds including prodelphinidin dimer B, procyanidin dimer, and quercetin hexoside isomer were identified from F-II. According to Table 1, compound **4** (RT = 2.56 min) showed the molecular ion ([M−H]^−^) at m/z of 593.1306 and was a monomeric unit of catechin and gallocatechin. The fragment ion m/z 407.0770 was due to a retro-Diels-Alder (RDA) fission of ring C and further elimination of a molecule of water, while the ion m/z 289.0715 was after a quinone-methide (QM) cleavage of the interflavan bond [24,25]. Compound **5** (RT = 4.53) showed a molecular ion ([M−H]^−^) at m/z of 577.1346 was identified as a procyanidin dimer which was a monomeric unit of catechin and epicatechin [26,27]. The produced two fragments at m/z of 407.07732 and 289.07161 were the same with that of prodelphinidin dimer B [25]. Compound **6** (RT = 4.53) had a ion [M−H]^−^ at m/z 463.0875 and yielded fragment ions at m/z 301.0336 and 300.0268 corresponding to losses of a hexose moiety [28]. Therefore, compound **6** was assigned as quercetin-3-O-glucoside [27].

As shown in Table 1, the main polyphenol compound in F-III was procyanidin dimer. Both compound **7** (RT = 4.91) and compound **8** (RT = 5.42) with the molecular ion ([M−H]^−^) at m/z of 577.1347 were identified as a procyanidin dimer.

### 2.2. Antioxidant Activity

#### 2.2.1. ABTS^+^ Radical Scavenging Ability of Polyphenol Fractions

**Figure 2:**
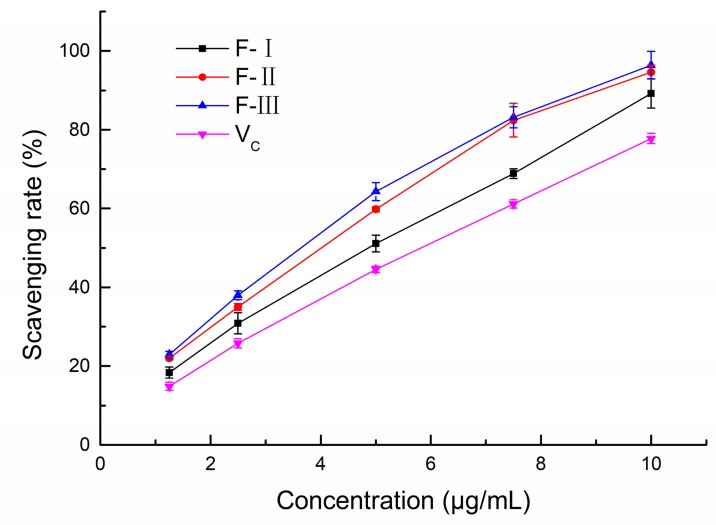
The capacities of three polyphenol fractions and vitamin C on 2,2′-azino-bis-3-ethylbenzthiazoline-6-sulphonic acid (ABTS^+^) radicals.

The 2,2′-azino-bis-3-ethylbenzthiazoline-6-sulphonic acid (ABTS^+^) radical scavenging abilities of the three polyphenol fractions from lotus seed epicarp (F-I, F-II, and F-III) are shown in Figure 2. Compared to V_c_, the ABTS^+^ radical scavenging capacities of the three polyphenol fractions were higher than that of V_c_ (*p* < 0.05). The IC_50_ values of F-I, F-II, and F-III were 5.04, 4.22, and 3.96 μg/mL, respectively. The result was lower than that of purified polyphenols from lotus seed epicarp (45.27 μg/mL) reported by Liu, Ma, Ibrahim, Li, Yang and Huang [17]. The better ABTS^+^ radical scavenging capacities of our polyphenol fractions might be due to the synergistic effect of various polyphenols. Generally, the ABTS^+^ radical scavenging capacities increased in the order of: F-III > F-II > F-I. Furthermore, at the concentration of 10 μg/mL, the ABTS^+^ radical scavenging rates of F-I, F-II, and F-III were 89.16%, 94.60%, and 96.38%, respectively. Our results indicate that the three polyphenol fractions from lotus seed epicarp had potential anti-radical activity on the ABTS^+^ radical.

#### 2.2.2. DPPH radical scavenging ability of polyphenol fractions

**Figure 3:**
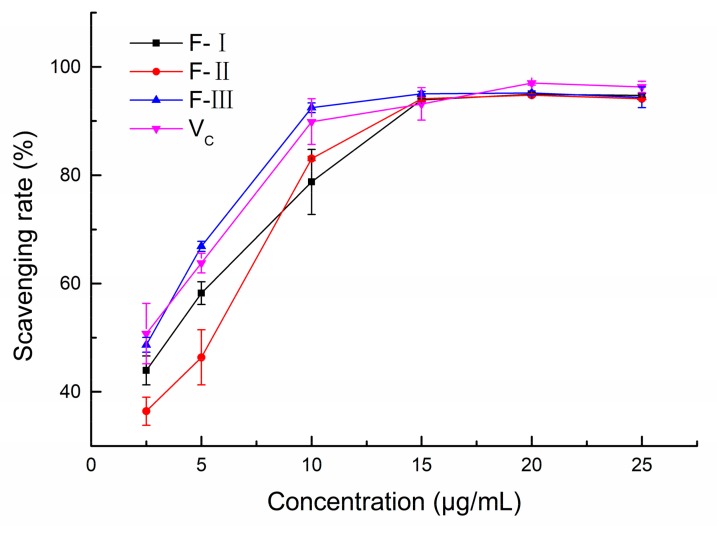
The capacities of three polyphenol fractions and vitamin C on 1,1-diphenyl-2-picryl-hydrazyl (DPPH) free radicals.

The 1,1-diphenyl-2-picryl-hydrazyl (DPPH) radical scavenging abilities of F-I, F-II, F-III and V_c_ are shown in Figure 3. The DPPH radical scavenging rates of the three polyphenol fractions firstly increased to the peak rate at the concentration of 15 μg/mL, and then the scavenging rates decreased steadily. F-III had a better anti-radical activity on DPPH than that of V_c_, whereas the DPPH radical scavenging rates of F-I and F-II were lower than that of V_c_ at the same concentration. The IC_50_ values of F-I, F-II, and F-III were 3.50, 4.75, and 2.26 μg/mL, respectively. The DPPH radical scavenging activity increased in the order of: F-III > F-I > F-II. Moreover, at a concentration of 15 μg/mL, the DPPH radical scavenging rates of F-I, F-II, and F-III reached 93.90%, 94.11%, and 95.03%, respectively. Our results thus indicated that the three polyphenol fractions from lotus seed epicarp had positive scavenging capacities on the DPPH radical.

#### 2.2.3. FRAP of Polyphenol Fractions

**Figure 4:**
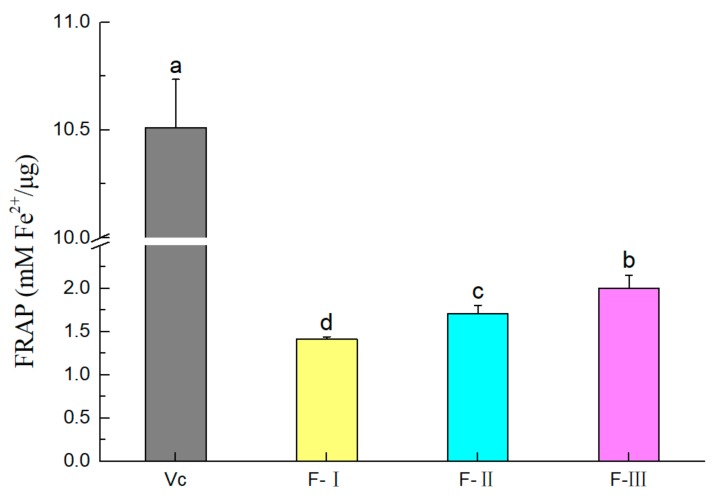
The ferric ion reducing antioxidant power (FRAP) abilities of three polyphenol fractions and vitamin C. Different letters (a–d) indicate significant differences (*p* < 0.05).

The ferric ion reducing antioxidant power (FRAP) of the three polyphenol fractions are shown in Figure 4. The FRAP values of F-I, F-II, and F-III were 1.41, 1.70, and 2.00 mMFe^2+^/µg, respectively. The results indicated that the FRAP of the polyphenol fractions increased in the order of: F-III > F-II > F-I (*p* < 0.05). The FRAP values were higher than other reported polyphenols [29,30]. Our results suggest that three polyphenol fractions from lotus seed epicarp had high FRAP levels.

The antioxidant activities of polyphenols from lotus seed epicarp were measured using FRAP assay as well as DPPH and ABTS^+^ radicals scavenging methods. These methods were widely used to measure antioxidant activity of natural compounds in vitro [31]. These tests have indicated different mechanisms of antioxidant action. The ABTS^+^ assay is superior to the DPPH assay when applied to samples containing hydrophilic, lipophilic, and highly pigmented antioxidant compounds [32]. We found that the three polyphenol fractions from lotus seed epicarp had strong antioxidant activities in vitro. Similar results were also reported about sweet potato leaf polyphenols [33]. In addition, there were significant differences among the three polyphenol fractions in the antioxidant activity assays. The F-III had the highest antioxidant activity, which may have been caused by the higher level of procyanidin dimer in F-III. Similar results have also been reported in that the procyanidin dimer is the most active free radical scavenging polyphenol [34,35].

## 3. Materials and Methods

### 3.1. Materials and Chemicals

Dried lotus seed epicarp (at full ripening stage with a water content of 10.3%) was from a local farm in HuNan province and then ground into powder, sieved through 100 meshes, and stored at −20 °C for further study.

AB-8 macroporous resin was purchased from Solarbio Co. (Beijing, China). Sephadex LH-20 was purchased from GE Healthcare Co. (Uppsala, Sweden). DPPH radical, ABTS^+^ radical, trolox and Folin–Ciocalteu reagent were purchased from Shanghai yuanye Bio-Technology Co., Ltd (Shanghai, China). Analytically pure anhydrous sodium carbonate, ascorbic acid, gallic acid monohydrate, sodium chloride, and potassium persulfate were obtained from Sinopharm Chemical Reagent Co., Ltd. (Beijing, China). Acetonitrile, methanol, and formic acid of chromatographic grade were purchased from Tedia (Shanghai, China).

### 3.2. Preparation of Crude Polyphenols from Lotus Seed Epicarp

Lotus seed epicarp powder was soaked in 60% ethanol solution at 1:20 ratio (w/v) in a 50 °C water bath for 90 min. The resulting solution was concentrated and lyophilized for further tests.

### 3.3. Determination of Total Phenolic Contents

Total phenolic content (TPC) was measured by a reported method [36]. Crude polyphenols from lotus seed epicarp was dissolved in ddH_2_O to a concentration of 1 mg/mL. Gallic acid standards or samples (20 μL) were incubated at room temperature for 1 min with 100 μL of 10-fold diluted Folin–Ciocalteu reagent in 96-well plates followed by reaction with 80 μL of 75 g/L Na_2_CO_3_ for 30 min and then absorbance at 765 nm was determined. All samples were measured in triplicate.

### 3.4. Gel Filtration System Purification of Lotus Seed Epicarp Polyphenols

Crude polyphenols from lotus seed epicarp was dissolved in ddH_2_O to a concentration of 5 mg/mL. After centrifugation at 10,000× *g* for 5 min, the supernatant was applied to a column of AB-8 macroporous resin. The column was eluted with ddH_2_O as mobile phase for 5 BV, and then followed by elution with 50% ethanol solution at a flow of 2 mL/min. The effluent was collected and subsequently dried with a rotary evaporator and lyophilizer.

The resulting purified polyphenols were loaded onto Sephadex LH-20 gel filtration chromatography column as described by Liu, Ma, Ibrahim, Li, Yang and Huang [17]. In short, the polyphenols were dissolved in methanol to a concentration of 50 mg/mL and centrifuged at 12,000× *g* for 5 min, and then the supernatant was separated with a Sephadex LH-20 gel filtration chromatography column which was eluted with methanol at a flow of 0.5 mL/min. Each fraction of 8 mL was collected. Elution curves were obtained by measuring absorbance at 280 nm using an on-line spectrophotometer. The three fractions were collected and further analyzed by ultra performance liquid chromatography tandem mass spectrometry (UPLC-MS).

### 3.5. UPLC-MS Analysis of Lotus Seed Epicarp Polyphenols

For RP-HPLC separations a Waters 2690 system (Waters Corporation, Milford, USA) equipped with an automatic sample injector was used. Separations were performed at 0.4 mL/min with eluent A consisted of 0.1% acetic acid in milli-Q water (*v*/*v*); eluent B of acetonitrile. The sample was filtered through a 0.22 mm syringe filter and injected into Acquity UPLC BEH C18 (2.1 mm × 100 mm, 1.7 μm) column. LC/UV traces were recorded on-line with a Waters 2690 PDA detector with detection at 280 nm. The elution program were as follows: 0–8 min, 5% B–35% B, 8–9 min, 35% B–50% B, 9–11 min, 50%–80% B, 11–12 min, 80% B–5% B. Mass spectrometry conditions: ESI^−^ ion source, spectra were recorded over the mass/charge (m/z) range 50–1000; capillary voltage: 2.5 kV, ion source temperature: 135 °C, solvent removal temperature: 350 °C, solvent removal gas velocity: 600 L/h, cone hole gas flow rate: 50 L/h, collision energy: 20~30 eV. Capillary temperature was 365 °C, and pressure of ESI nebulizing gas (N_2_) was 275.79 kPa (40 psi); flow rate of drying gas (N_2_) was 10.00 L/min.

### 3.6. Antioxidant Assay of Lotus Seed Epicarp Polyphenols 

#### 3.6.1. DPPH Radical Scavenging Activity

The capacity of lotus seed epicarp polyphenols (LSEP) purified by Sephadex LH-20 to scavenge DPPH free radicals was assessed as described by Chen, Ma and Kitts [36]. LSEP purified by Sephadex LH-20 were dissolved in ddH_2_O and diluted in methanol to 100 µg/mL. Samples were incubated with 0.1 mM DPPH in the final volume of 200 μL at room temperature for 10 min in darkness and then absorbance at 519 nm was determined. V_c_ was used as standard. The percentage inhibition of the DPPH free radical was calculated as % Inhibition = (A_c_−A_s_)/(A_c_−A_b_) × 100, where A_c_ = absorbance of 0.1 mM DPPH alone in methanol, A_s_ = absorbance of 0.1 mM DPPH with sample in methanol, A_b_ = absorbance of methanol solvent in absence of DPPH and sample. The IC_50_ values were calculated using OriginPro software (OriginLab Corporation, Northampton, MA, USA).

#### 3.6.2. ABTS^+^ Radical Scavenging Activity

Assays of ABTS proceeded essentially as described by Chen et al. [36]. ABTS (5 mL, 7 mM) was mixed with K_2_S_2_O_8_ (88 μL of 140 mM) overnight at room temperature in the dark. The stock ABTS solution (0.6 mL) was diluted with ddH_2_O (40 mL) and absorbance adjusted to 0.7 at 734 nm to make the ABTS working solution. The samples were prepared as the methods mentioned above. Samples of 20 μL in total were reacted with ABTS working solution (180 μL) at room temperature in darkness for 10 min, before recording absorbance readings taken at 734 nm by spectrophotometry. V_c_ was used as standard. The percentage inhibition of the ABTS^+^ radical was calculated as % Inhibition = (A_c_−A_s_)/(A_c_−A_b_) ×100, where A_c_ = absorbance of ABTS^+^ alone in methanol, A_s_ = absorbance of ABTS^+^ with sample in methanol, A_b_ = absorbance of methanol solvent in absence of ABTS^+^ and sample. The IC_50_ values were calculated using OriginPro software (OriginLab Corporation, Northampton, MA, USA).

#### 3.6.3. Ferric Ion Reducing Antioxidant Power (FRAP) Assay

The FRAP was evaluated using the reported method [37,38]. In brief, the FRAP working solution was prepared to mix acetate buffer (pH 3.6, 300 mM), 2,4,6-tris (2-pyridyl)-s-triazime (TPTZ) solution (10 mM) and FeCl_3_ solution (20 mM) with a volume ratio 10:1:1. Afterwards, 5 μL sample solution and 180 μL FRAP working solution was mixed with reaction at room temperature for 6 min. The absorbance was determined at 593 nm. Different concentrations of FeSO_4_ were used as standards. The concentration of FeSO_4_ (mM Fe^2+^/μg) was indirectly expressed as with equivalent antioxidant activity of sample.

### 3.7. Statistical Analysis

The result was expressed as mean values and standard deviation (SD). The variance was determined by the one-way analysis of variance (ANOVA) and differences between the samples were measured by Duncan’s test (0.05).

## 4. Conclusions

In this study, we separated polyphenols from lotus seed epicarp into three fractions—F-I, F-II, and F-III. Six kinds of polyphenol compounds in the three fractions were identified by UPLC-MI-TOF-MS. Cyanidin-3-*O*-glucoside, procyanidin trimer, and phlorizin were identified in F-I, prodelphinidin dimer B, procyanidin dimer, and quercetin hexoside isomer were found in F-II, whereas only procyanidin dimer was identified in F-III. We found that the three polyphenol fractions from lotus seed epicarp had strong FRAP values as well as positive scavenging abilities against DPPH and ABTS radicals. Moreover, F-III had the best antioxidant activity among the three polyphenol fractions. The polyphenols from lotus seed epicarp might be a good candidate as a natural food additive. However, the application of polyphenols from lotus seed epicarp in food preservation remains to be further studied.

## Figures and Tables

**Table 1:** Identification of compounds in three polyphenol fractions from lotus seed epicarp.

Compound	Fraction	Retention Time (min)	[M−H]^−^ (m/z)	Typical MS/MS Ions (m/z)	Formula	Identification
1	F-I	4.68	449.1087	287.0558	C_21_H_21_O_11_	Cyanidin-3-*O*-glucoside
2	F-I	5.15	865.1975	449.1088, 287.0557	C_45_H_38_O_18_	Procyanidin trimer
3	F-I	7.75	435.1293	273.0761	C_21_H_24_O_10_	Phlorizin
4	F-II	2.56	593.1306	407.0770, 289.0715	C_30_H_26_O_13_	Prodelphinidin dimer B
5	F-II	4.53	577.1346	407.0770, 289.0715	C_30_H_26_O_12_	Procyanidin dimer
6	F-II	6.23	463.0875	301.0336, 300.0268	C_21_H_20_O_12_	Quercetin hexoside isomer
7	F-III	4.91	577.1347	407.0770, 289.0711	C_30_H_26_O_12_	Procyanidin dimer
8	F-III	5.42	577.1347	407.0770, 289.0713	C_30_H_26_O_12_	Procyanidin dimer
